# Effects of Photons Irradiation on ^18^F-FET and ^18^F-DOPA Uptake by T98G Glioblastoma Cells

**DOI:** 10.3389/fnins.2020.589924

**Published:** 2020-11-13

**Authors:** Francesca Pasi, Marco G. Persico, Manuela Marenco, Martina Vigorito, Angelica Facoetti, Marina Hodolic, Rosanna Nano, Giorgio Cavenaghi, Lorenzo Lodola, Carlo Aprile

**Affiliations:** ^1^Oncology Unit, Fondazione IRCCS Policlinico San Matteo, Pavia, Italy; ^2^Department of Biology and Biotechnology “Lazzaro Spallanzani”, University of Pavia, Pavia, Italy; ^3^University School for Advanced Studies IUSS Pavia, Pavia, Italy; ^4^Nuclear Medicine Unit, Fondazione IRCCS Policlinico San Matteo, Pavia, Italy; ^5^CNAO National Centre for Oncological Hadrontherapy, Pavia, Italy; ^6^Nuclear Medicine Research Department, IASON, Graz, Austria; ^7^Nuclear Medicine Department, Faculty of Medicine and Dentistry, Palackı University Olomouc, Olomouc, Czechia

**Keywords:** photon irradiation, ^18^F-DOPA, glioblastoma, radionecrosis, T98G cells, ^18^F-FET

## Abstract

The differential diagnosis between brain tumors recurrence and early neuroinflammation or late radionecrosis is still an unsolved problem. The new emerging magnetic resonance imaging, computed tomography, and positron emission tomography diagnostic modalities still lack sufficient accuracy. In the last years, a great effort has been made to develop radiotracers able to detect specific altered metabolic pathways or tumor receptor markers. Our research project aims to evaluate irradiation effects on radiopharmaceutical uptake and compare the kinetic of the fluorinate tracers. T98G glioblastoma cells were irradiated at doses of 2, 10, and 20 Gy with photons, and ^18^F-DOPA and ^18^F-FET tracer uptake was evaluated. Activity and cell viability at different incubation times were measured. ^18^F-FET and ^18^F-DOPA are accumulated via the LAT-1 transporter, but ^18^F-DOPA is further incorporated, whereas ^18^F-FET is not metabolized. Therefore, time-activity curves (TACs) tend to plateau with ^18^F-DOPA and to a rapid washout with ^18^F-FET. After irradiation, ^18^F-DOPA TAC resembles the ^18^F-FET pattern. ^18^F-DOPA activity peak we observed at 20 min might be fictitious, because earlier time points have not been evaluated, and a higher activity peak before 20 min cannot be excluded. In addition, the activity retained in the irradiated cells remains higher in comparison to the sham ones at all time points investigated. This aspect is similar in the ^18^F-FET TAC but less evident. Therefore, we can hypothesize the presence of a second intracellular compartment in addition to the amino acidic pool one governed by LAT-1, which could explain the progressive accumulation of ^18^F-DOPA in unirradiated cells.

## Introduction

Glioblastoma multiforme (GBM) represents the most common and aggressive primary malignancy form, with a poor prognosis (survival is about 12–18 months). Robust neovascularization, massive glioma cell invasiveness within the whole brain parenchyma, and resistance to conventional therapies characterize GBM evolution, flowing into frequent recurrence and relapse (Gilbert, 2011).

Diagnosis of GBM is based on magnetic resonance imaging, computed tomography, and positron emission tomography (PET) imaging. To date, the imaging protocol is still controversial and lacks of accurate tools, because of the abundant rate of peritumoral necrosis and inflammation that smooth the abilities to closely define the tumor boundaries and affect the efficiency of early diagnosis (Petrujkiæa et al., 2019).

Moreover, the GBM multimodal treatment provides the combination of surgical resection, radiotherapy, and chemotherapy that lead to a local inflammation process, the so-called radionecrosis phenomenon. Radionecrosis strongly affects the ability of rapid and unambiguous relapse discrimination, compromising the treatment strategies and options during the follow-up of the patient (Carlsson et al., 2014).

Recent advances in imaging techniques have opened endless opportunities for molecular diagnostic and therapeutic procedures. Molecular imaging could help in early detection, characterization, and “real-time” monitoring of various diseases. Regarding cancers, it could be an excellent tool to investigate treatment’s efficacy, allowing areas of interest to be monitored during therapy and supporting the follow-up process. PET has proven to be a useful imaging modality in the distinction between benign and malignant lesions and in the assessment of peritumoral areas (Graves et al., 2007; Buroni et al., 2015; Frosina, 2016).

^18^F-FDG is the most common PET radiopharmaceutical, able to detect the upper glucose uptake in the tumor region. Unfortunately, ^18^F-FDG is not applicable to detect brain tumors, such as GBM, due to the physiological high glucose metabolism rate in the brain (Nanni et al., 2010).

Hence, in the last years, a great effort has been made to develop alternative metabolic or receptorial radiotracers, able to detect specific altered metabolic pathways or tumor receptor markers.

^18^F-choline (^18^F-FCH), a fluorinated phosphatidylcholine precursor, is approved for diagnosis of prostate and hepatocellular carcinomas, and its use is currently extending to other cancers, including GBM. Our previous research demonstrated that ^18^F-FCH is able to trace tumor behavior in terms of higher uptake for increased doses of radiation treatment (Pasi et al., 2017).

Nevertheless, ^18^F-FCH has shown several limitations in the diagnosis of those tumors characterized by a high inflammatory component, therefore reducing the PET clinical impact (Spaeth et al., 2006). To overcome these crucial issues, amino acidic radiopharmaceuticals, such as ^18^F-ethyl-L-tyrosine (^18^F-FET) and 6-^18^F-L-3,4-dihydroxyphenylalanine (^18^F-DOPA), have been developed. The overexpression of amino acidic transporters is a peculiar feature of cancer cells, because of the significant increase in protein synthesis and could be a reason of their future use in diagnostics (Stadlbauer et al., 2008).

^18^F-FET is a tyrosine analog tracer that is internalized mostly by L-type-amino acid methionine Na + -independent transporter 1 (LAT-1), expressed in brain endothelial cells and in tumor cells, including GBM.

Another amino acidic tracer is the ^18^F-DOPA, an analog of L-DOPA, carried in the cytoplasm of cancer cells by the LAT system, as for ^18^F-FET. ^18^F-DOPA was initially employed to evaluate presynaptic dopaminergic neuronal function in patients with movement disorders as precursor of dopamine with accumulation in the basal ganglia and without significant accumulation in the brain parenchyma. The oncological interest of ^18^F-DOPA arises from the incidental discovery of a G-II oligoastrocytoma in a patient with suspicious Parkinson disease (Heiss et al., 1996).

In this article, we focused on comparing ^18^F-FET and ^18^F-DOPA, investigating their kinetic uptake in GBM cells in basal condition and after crescent doses of photon irradiation.

As discussed, the differential diagnosis between brain tumor recurrence and early neuroinflammation or late radionecrosis is still an unsolved problem. Our research project aims to evaluate the cellular response of T98G glioblastoma cells, chosen because of its radioresistant and aggressive features, by isolating it from the influence of the microenvironment. Although this approach is in part limiting of what occurs *in vivo*, it allows identifying the alterations within the tumor cell in basal conditions and after irradiation that could be useful in the clinic. In fact, it is of great importance for physicians to consider the different kinetics pathways of uptake concerning the two radiopharmaceuticals, in order to define the one of major interests, and the behavior of the different types of cells after irradiation. This is the first step of a larger project, which consists of the study of neoplastic, endothelial, microglia, and tumor cells incubated with medium harvested from irradiated ones, containing inflammatory and growth factors, cytokines, receptor ligands, and other factors that could contribute to the development of radiation necrosis. Although this system limits translatability due to the difference in tissue attenuation *in vivo* compared to cell cultures (Paget et al., 2019), our aim is to study the influence of ionizing radiation on the transport mechanism of labeled amino acids, namely, ^18^F-FET and ^18^F-DOPA, used in PET imaging and therefore on tumor cell uptake by discriminating early/late recurrence radionecrosis.

## Materials and Methods

### Cell Culture

Human glioblastoma T98G cells were obtained from the European Collection of Cell Cultures (Porton Down, Salisbury, United Kingdom). T98G cells were cultured in eagle minimum essential medium (EMEM; Euroclone SpA, MI, Italy) supplemented with 10% fetal bovine serum (Sigma–Aldrich, St. Louis, MO, United States), 100 U/mL penicillin/streptomycin (Euroclone SpA, MI, Italy), 2 mM L-glutamine (Euroclone SpA, MI, Italy), and 0.01% sodium pyruvate (Sigma–Aldrich, St. Louis, MO, United States) at 37°C in an atmosphere of 5% CO_2_. Stock cultures were maintained in exponential growth as monolayers in 75-cm^2^ Corning plastic tissue-culture flasks (VWR International PBI Srl, MI, Italy).

### Irradiation Treatments

**FIGURE 1:**
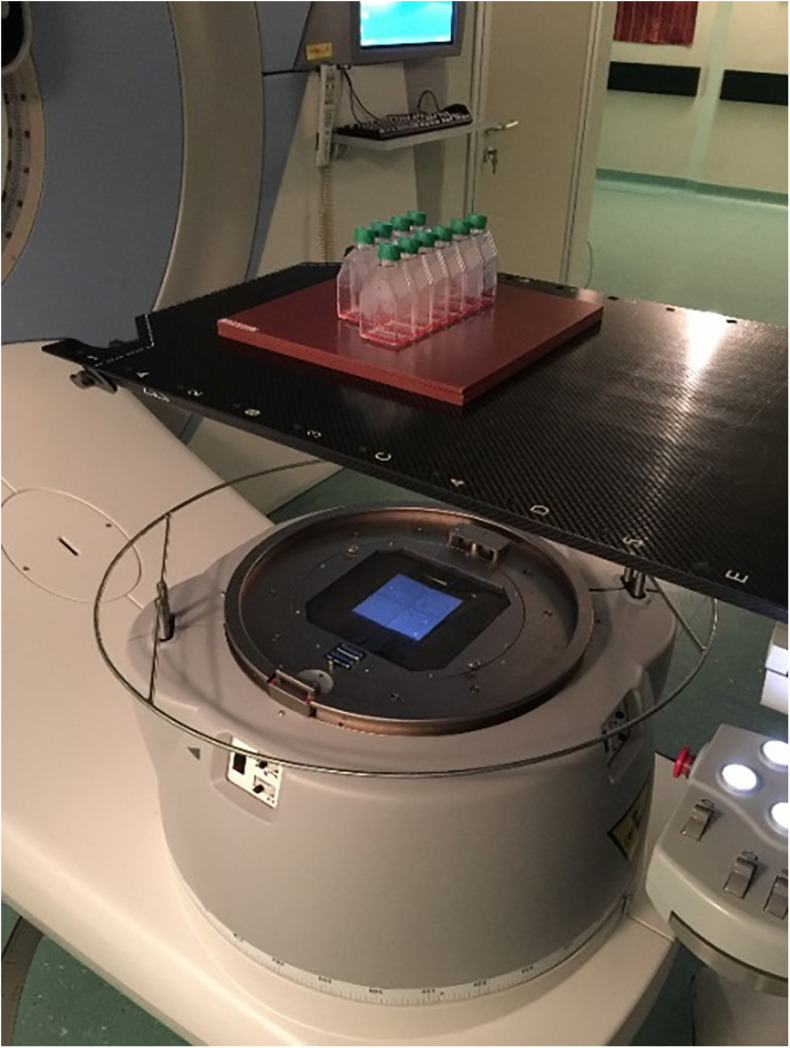
Experimental setup used for T98G cell irradiations at radiotherapy unit.

Cells were irradiated at doses of 2, 10, and 20 Gy with photons at room temperature using a LINAC at 6 MeV (ELEKTA Synergy; Radiotherapy Unit, Fondazione IRCCS Policlinico San Matteo, Pavia, Italy) with a dose rate of 3 Gy/min. The flasks containing the cells were placed vertically at the isocenter, and a 5-mm-thick plastic sheet was placed below the flask surface to allow dose build-up (Figure 1). Sham-irradiated cells (0 Gy) were performed as control. An hour before irradiation, the medium was removed from the flasks, and fresh medium was added to the cells. Cells were replaced in incubator at 37°C after irradiation treatment.

### Radiopharmaceutical Incubation

^18^F-DOPA (IASOdopa^®^) and ^18^F-FET (IASOglio^®^) were obtained from IASON GmbH (Graz, Austria). T98G cells grew adherent to the plastic surface at 37°C in 5% CO_2_ in complete medium.

Samples with 2 × 10^5^ cells per flask were irradiated at room temperature 20 h after seeding. A total of 36 h post-irradiation, tracer uptake was evaluated after addition of 100 kBq (100 μL) of to each flask with 2 mL of medium.

Activity in the adherent cells and the number of surviving cells and their viability at different incubation times (20, 40, 60, 80, and 120 min) were measured. Tracer incubation was done in complete medium. Control samples underwent the same procedure as other samples, but they were incubated with 100 μL of saline instead of a radiotracer.

### Cell Kinetic Studies and Uptake Evaluation

The cellular radiotracer uptake was determined with a 3 × 3” NaI(Tl) pinhole 16 × 40-mm gamma counter (Raytest, Straubenhardt, Germany). All measurements were carried out under the same counting position, along with a standardized source to verify the counter’s performance, and the data were corrected for background and decay. Total radioactivity was counted when the radiotracer was added to the medium in each flask (time 0). After 20, 40, 60, 90, and 120 min from time 0, the medium was harvested, the cells were rapidly washed three times with 1 mL of phosphate-buffered saline, and radiopharmaceutical uptake for each sample was assessed. The uptake measurements are expressed as the percentage of the administered dose of tracer per 2 × 10^5^ cells after correction for negative control uptake (flasks containing no cells with complete medium and incubated with radiopharmaceutical). Damaged cells were evaluated with propidium iodide (PI) and annexin V fluorescent staining as previously described (Pasi et al., 2017).

### Statistical Analysis

*In vitro* experiments were conducted in duplicate for each experimental point and repeated twice the full experiment. All values are expressed as mean values with confidence interval (95% CI). The uptake of radiotracer is represented as a function of the incubation period; all values are shown in figures as a percentage of the administered dose per 2 × 10^5^ cells (mean ± 95% CI). Therefore, if error bars on the *y* axis do not overlap, the two points are considered significantly different (*p* < 0.05).

## Results

Results were expressed as added dose (%) on 2 × 10^5^ cells, corrected for decay and for non-specific binding. Sham-irradiated cells (0 Gy) were considered as control.

**FIGURE 2:**
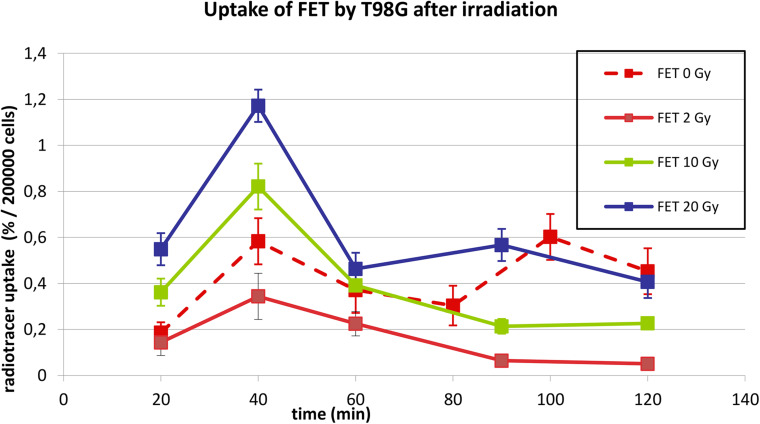
Uptake of ^18^F-FET in T98G cells in basal condition and after photon irradiation with 2, 10, and 20 Gy.

Figure 2 shows the uptake of ^18^F-FET by T98G cells in basal conditions and after irradiation with photons at doses of 2, 10, and 20 Gy. In basal condition, the uptake increased up to 40 min and then decreased up to 80 min. Subsequently, it increased again to 100 min and then decreased.

After irradiation, the curves showed a dose-dependent uptake with a similar trend, showing a peak at 40 min. After 60 min, a plateau was reached for each irradiation dose.

**FIGURE 3:**
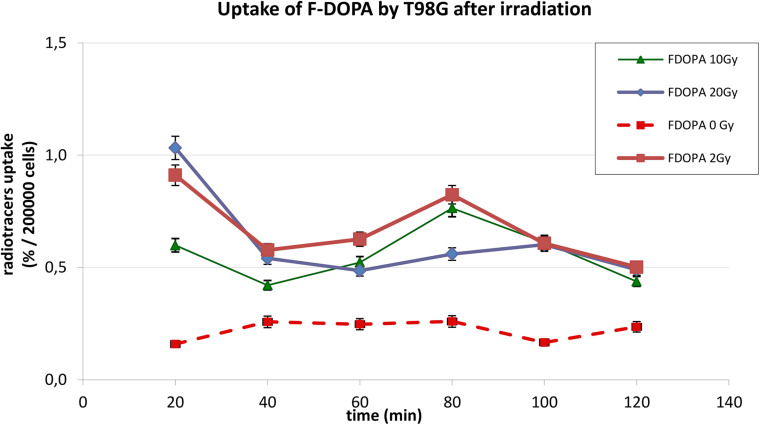
Uptake of ^18^F-DOPA in T98G cells in basal condition and after photon irradiation with 2, 10, and 20 Gy.

The behavior of ^18^F-DOPA was different, as present on Figure 3; in basal conditions, T98G cells reached a maximal uptake 40 min after ^18^F-DOPA addition, afterward reaching a plateau. In photon-irradiated cells, the kinetic pattern changes dramatically; absolute uptake of ^18^F-DOPA increases more than two or three times after irradiation, and a peak activity was observable first at 20 min, and we cannot rule out earlier higher activity before 20 min, followed by a rapid washout and by a further reuptake at 80 min.

In Figures 2, 3, statistical significance (*p* < 0.05) can be evaluated with the overlapping or not of error bars on *y* axis. Indeed, they represent the 95% CI of the single experimental point; e.g., each value at 20 to 40 min about ^18^F-FET uptake (Figure 2) is significantly different from each other (0–2–10–20 Gy); in the same time series at 20 Gy, experimental point at 40 min is statistically different from the others. Analogous evaluation can be drawn for all time series and in Figure 3 as well.

**FIGURE 4:**
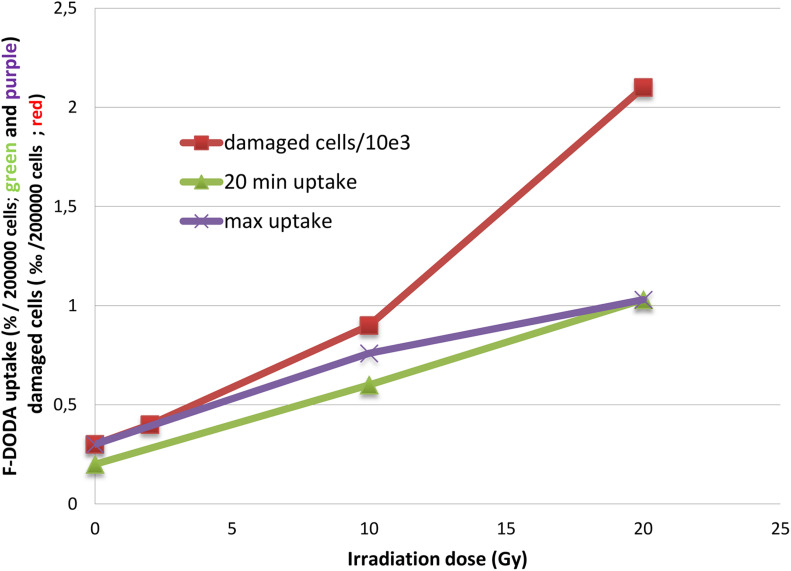
Trend of ^18^F-DOPA uptake after 20 min and at the peak activity compared to the number of damaged cells.

After irradiation, as with other radiopharmaceuticals we previously reported (Pasi et al., 2017), the net uptake normalized to 2 × 10^5^ cells appears to be increased, but most noticeable is the striking change of the TAC pattern. We observed that the percentage of damaged cells 35 h after irradiation as shown by PI and annexin V fluorescent staining increases in an exponential pattern (4% at 2 Gy, 9% at 10 Gy and 21% at 20% vs. 3% of control), whereas the ^18^F-DOPA uptake at peak value increases in a linear one (Figure 4).

## Discussion

Both FET and DOPA share the common characteristic to increase their uptake in T98G as response to radiation. We and other authors either for different labeled substrates and various cell lines have previously reported this apparent paradoxical behavior (Cheon et al., 2007; Pasi et al., 2017).

The main difference between the two tracers is related to the shape of TAC. In fact, ^18^F-FET TAC shows only a modification of the peak value, whereas with ^18^F-DOPA, major shape changes are observable. Sham cells show a maximal ^18^F-DOPA uptake at 40 min, plateaus during the observation time, whereas an early peak can be found after irradiation.

The irradiation system used in this work is LINAC, the medical linear accelerator used in clinic. It is used to mimic the cellular effect induced in cells by ionizing radiation at the same doses and energies used for therapy. It is the most used in clinic and in clinical research on cell cultures (Alexiou et al., 2019). The effect of treatment was evaluated after 36 h from irradiation. T98G cells seem not to respond to ionizing radiation after 24 h at a dose of 20 Gy (Murad et al., 2018). We considered more time for an effect to be appreciable compared to control.

Many factors have been postulated to explain modifications of tumor cells to ionizing radiation, which paradoxically can promote metastasis and invasion of cancer cells (Lee et al., 2017). ^18^F-FET does not participate in specific metabolic pathways such as catecholamine metabolism. The influence of catecholamine metabolism and melanin synthesis on ^18^F-FET uptake in tumors as well as in other tissues appears to be negligible.

Although ^18^F-FET is not incorporated into proteins, uptake by tumor cells is stereospecific and mediated by amino-acid transporters, whereas for methionine a 15% protein incorporation after 2-h incubation time was noted (Langen et al., 2017). On the other side, Habermeier and colleagues hypothesized an intracellular metabolism leading to another impermeable derivative trapped within glioma cell, suggesting an asymmetry of intracellular and extracellular recognition by LAT-1 (Habermeier et al., 2015).

^18^F-FET uptake decreases after a peak of activity (Pasi et al., 2017). This pattern resembles that observed *in vivo* with PET (Galldiks et al., 2012) where the peak time is related to tumor aggressiveness followed by a steep decrease.

The increased uptake induced by irradiation has been previously described for ^18^F-FET in T98G (Pasi et al., 2017) and MCF7 cell lines (Cheon et al., 2007). Many factors could be involved in this apparent paradox including up-regulation of the amino-acid transporter LAT-1 (Habermeier et al., 2015) and selection of more aggressive radioresistant clones (Pasi et al., 2017). The role of p53 seems less plausible in T98G p53mt (Van Meir et al., 1994); in fact, ^18^F-FET accumulation in MCF7 cell line increases independently of p53 status. Therefore, p53 does not influence the LAT-1 mechanism, whereas a clear influence of p53wt and p53mt on the ^18^F-FDG and ^18^F-FLT incorporation after beta and gamma radiation has been observed (Cheon et al., 2007).

As mentioned previously, in our *in vitro* model, ^18^F-DOPA reaches its maximum uptake after 40 min of incubation and plateaus up to 80 min before decreasing. This behavior does not support a simple transport uptake mechanism but suggests a further trapping mechanism.

The mechanism of ^18^F-DOPA accumulation in glioma has not been thoroughly elucidated, even if up-regulation of its transporter seems to play a key role. The LAT-1, a sodium-independent neutral amino-acid transporter, facilitates L-DOPA transport in endothelial cells of brain capillaries and in kidney epithelial cells. LAT-1 up-regulation correlates with tumor grade and a reduction of uptake with response to treatment and a similar consideration can be done for ^18^F-FET.

Nonetheless, the intensity of ^18^F-DOPA uptake is not directly correlated with the level of LAT-1 expression. A minimal expression of LAT1 is required for ^18^F-DOPA uptake, but this amount is not linearly related to the histochemical score of LAT-1 expression, suggesting that the balance between influx and efflux is not sufficient to understand the mechanism of uptake and retention (Dadone-Montaudié et al., 2017).

These results are partially contradictory with the Youland and colleagues’ report (Youland et al., 2013), who found *in vivo* a linear relationship between standardized uptake value (SUV) and LAT-1 expression. GBM cell lines with low LAT-1 expression had significantly less ^3^H-L-DOPA uptake compared to cell lines with readily detectable LAT-1.

In addition, *in vitro* knockdown of LAT-1 reduces ^3^H-L-DOPA uptake in human glioma lines T98G and GBM28. Nevertheless, despite robust knockdown, the uptake is only reduced by approximately 50 to 70%, suggesting that other mechanisms account for tracer incorporation and differences related to brain lesions. Unfortunately, in Youland and colleagues’ report, only one experimental time point is taken into account, i.e., 20 min after tracer addition, and the comparison of these data with our TAC is not possible.

Another difference between *in vivo* and *in vitro* model appears noticeable: ^18^F-DOPA competes with tyrosine for the LAT-mediated uptake. Tyrosine concentration in the medium used *in vitro* (EMEM) is 0.230 mmol/L, whereas in plasma it is 0.077 (Schmidt et al., 2016), indicating a competitor concentration twofold to threefold higher than *in vivo*.

The model works quite satisfactorily in brain gliomas (Schiepers et al., 2007; Wardak et al., 2014), where PET external detection is employed, but it does not take into account other signal sources as endothelial, infiltrating normal cells, vascularization, and metabolites such as L-3,4-dihydroxy-6-fluoro-3-O-methylphenylalanine (OMFD).

Retention after peak activity may be related to the presence of a second intracellular compartment, marked with a question point in the Schiepers and colleagues’ study, in addition to the intracellular amino acidic pool (Schiepers et al., 2007; Youland et al., 2013). There are no evidences if this secondary compartment could be attributed to storage or a metabolic pool.

Differently from ^18^F-FET, the TAC shape we observed *in vitro* is different from the one reported *in vivo* with PET. The ^18^F-DOPA pattern we observed does not appear to mimic the PET *in vivo* TACs. Schiepers and colleagues’ study (Schiepers et al., 2007) described an early steep ascent in tumor curve and a parallel cerebellum washout curve. On the contrary, striatum where metabolism is active leads to a plateau-shaped curve.

Ginet and colleagues, on the contrary, described different shapes of the PET TACs (Ginet et al., 2020). IDH mutation status and 1p/19q codeletion status seem to influence markedly the uptake rate and the residence time in the tumor of ^18^F-DOPA, whereas IDHwt GBM curve resembles ^18^F-FET curve with an early peak followed by a rapid washout. T98G, as many other glioma cell lines, does not display the IDH1^*R*132^ variant or the 1p/19q total deletion (Bleeker et al., 2009; Ichimura et al., 2009).

*In vivo* ^18^F-DOPA is partially metabolized in blood and by peripheral tissues as liver to OMFD (L-3,4-dihydroxy-6-fluoro-3-*O*-methylphenylalanine), whereas such metabolic way is negligible in the brain and absent in the tumor. OMFD is transported bidirectionally in the brain, both in striatum and in tumor, showing a significant affinity for tumor cells (Chopra, 2007; Haase et al., 2007). It cannot be distinguished *in vivo* from parent ^18^F-DOPA by external detection because it retains the F-label. ^18^F-DOPA and OMFD are incorporated in tumor cell via the LAT-1. In an *in vitro* model, the OMFD contribution is absent and hampers a more precise comparison with the ^18^F-DOPA kinetics described by PET studies, where the combined activities of ^18^F-DOPA and OMFD appear to move in and out of tumor contributing in the same way as the signal detection.

The fluorinated amine shows a metabolic behavior similar to non-labeled ones, even if fluorinated compounds may have different biological properties in comparison to parent compounds. Isosteric F-substitution at ring position 6 is the best substrate for L-DOPA decarboxylase (DDC) (Daidone et al., 2012). The L-DDC is a pyridoxal-5-phosphate–dependent enzyme participating in the biosynthesis of catecholamines, catalyzing the decarboxylation of L-DOPA to dopamine. In addition to the central nervous system, DDC is present in many peripheral organs such as liver, kidneys, gastrointestinal tract, and pancreas, in which the biological function is yet to be determined (Patsis et al., 2012). Surprisingly, approximately 20% of non-neuroendocrine tumors express DDC, even if it has been found in three glioblastoma specimens (Gazdar et al., 1988). Novel DDC splice variants have been detected in 40 tumor cell lines from 11 types of cancer, including brain tumors U-87 MG, U-251 MG, D54, H4, and SH-SY5Y. These protein isoforms might participate in metabolic processes having alternative functions (Adamopoulos et al., 2019). For example, HeLa and HTB14 (human glioblastoma) coexpress the non-neural full-length DDC mRNA and the alternative neural transcript lacking exon 3, but no enzymatic activity is detectable in the cellular extracts (Chalatsa et al., 2011). Nevertheless, T98G cells do not seem to express the gene coding DDC (Functional Annotation of the Mammalian Genome, 2012).

Randomly, mis-incorporation of ^18^F-DOPA into newly synthetized proteins is another aspect that has been neglected until now, and it has been described in patients assuming L-DOPA for therapeutic purpose. However, this behavior depends on the ratio of L-DOPA to L-tyrosine in the cell (Rodgers et al., 2006; Chan et al., 2012). Therefore, this hypothesis is weak because in culture medium ^18^F-DOPA is present in femtomolar concentration while tyrosine in millimolar concentration.

Regarding T98G cell line used in our test, there is no further information available that can support a hypothetic metabolic pathway. On the other hand, based on TAC shape, which indicates retention with late egress, a second intracellular compartment can be hypothesized.

After irradiation, the TAC shape is more similar to the ^18^F-FET one with an early peak activity followed by a sharp decrease; nonetheless, the latter experimental points are higher than in basal condition. The ^18^F-DOPA activity peak we observed at 20 min might be fictitious, because earlier time points have not been evaluated, and a higher activity peak before 20 min cannot be excluded. Noticeably, the damaged cell percentage increased in an exponential manner, whereas ^18^F-DOPA uptake at peak value increases linearly (Figure 4).

The effects of irradiation on LAT-1 expression in tumors and on ^18^F-DOPA uptake are unknown. In addition, the comparison with PET activity in patients submitted to radiotherapy is not immediate and can furnish only limited information. Chiaravalloti and colleagues reported *in vivo* a significantly higher SUV related to a smaller interval from RT, whereas this relationship was not demonstrable after chemotherapy (Chiaravalloti et al., 2015). They attributed this finding to a blood–brain barrier disruption and a subacute or delayed inflammatory process. This explanation is appropriate for external imaging but not in our model where the microenvironment influence is absent. In addition, it is not possible to discriminate in this group patients who were free of disease versus recurrence in a short time. On the contrary, Dadone and colleagues did not find any significant correlation between the interval from radiotherapy to PET scan in terms of SUV (Dadone-Montaudié et al., 2017).

Therefore, taking into account the TAC in non-irradiated and in the 10- and 20-Gy irradiated cells, we can hypothesize the presence of a second intracellular compartment in addition to the amino acidic pool one governed by LAT-1, which could explain the progressive accumulation of ^18^F-DOPA in non-radiated cells. Indeed, if the LAT-1 system were the unique mechanism responsible for ^18^F-DOPA uptake, we should have observed an uptake pattern similar to the ^18^F-FET one. There are no indications if this second compartment has a metabolic or storage function and if there is an exchange between the two compartments. Protein incorporation is less likely, and some relationship with DDC is not demonstrable.

Another mechanism different from LAT-1 plays a significant role in the ^18^F-DOPA incorporation with a temporal shift in comparison with LAT-1, whose robust knockdown diminishes but does not abolish uptake. Therefore, in basal condition, the plateau phase could represent the additive effect of LAT-1 and the other mechanism of uptake and/or retention. After irradiation, this second mechanism is diminished or unchanged, whereas LAT-1 is up-regulated; therefore, the descending tail of the TAC referable to LAT-1 activity can mask the contribution if any of this second hypothetical mechanism occurs.

The limitation of our experimental approach is related to the fact that an *in vitro* model cannot take into account many factors as the contribution of the microenvironment and labeled metabolites produced by non-tumor cells, which both provide signal detected by PET. In order to translate our results into preclinical application, physicians will have to consider the different kinetics pathways of uptake concerning the two radiopharmaceuticals. Further evaluation should be carried out to understand the underlying mechanism here hypothesized.

## Data Availability Statement

The original contributions presented in the study are included in the article/supplementary material, further inquiries can be directed to the corresponding author.

## Author Contributions

FP designed and performed the experiments, and wrote the manuscript. MP designed and performed the experiments, and supervised the findings of this work. MM carried out the experiments and wrote the manuscript. MV carried out the experiments. AF discussed the results and contributed to the final manuscript. MH provided the radiopharmaceuticals and contributed to the final version of the manuscript. RN contributed to the implementation of the research and helped supervise the project. GC supervised the project. LL contributed to the implementation of the research and worked on the manuscript. CA conceived of the presented idea and contributed to the design and implementation of the research, to the analysis of the results, and to the writing of the manuscript. All authors provided critical feedback and helped shape the research, analysis, and manuscript.

## Conflict of Interest

MH is Clinical Research Supervisor at IASON, Graz, Austria. The remaining authors declare that the research was conducted in the absence of any commercial or financial relationships that could be construed as a potential conflict of interest.
